# Impact of multimorbidity: acute morbidity, area of residency and use of health services across the life span in a region of south Europe

**DOI:** 10.1186/1471-2296-15-55

**Published:** 2014-03-26

**Authors:** Quintí Foguet-Boreu, Concepció Violan, Albert Roso-Llorach, Teresa Rodriguez-Blanco, Mariona Pons-Vigués, Miguel A Muñoz-Pérez, Enriqueta Pujol-Ribera, Jose M Valderas

**Affiliations:** 1 Institut Universitari d’Investigació en Atenció Primària Jordi Gol (IDIAP Jordi Gol), Gran Via Corts Catalanes, 587 àtic, 08007 Barcelona, Spain; 2 Universitat Autònoma de Barcelona, Plaza Cívica, Campus de la UAB, 08193 Bellaterra, Cerdanyola del Vallès, Spain; 3 Hospital de Campdevànol, Ctra. de Gombrèn, 20, 17530 Campdevànol, Spain; 4 Health Services & Policy Research Group, School of Medicine, University of Exeter, Exeter EX1 2LU, UK

**Keywords:** Multimorbidity, Chronic disease, Acute disease, Life-stage

## Abstract

**Background:**

Concurrent diseases, multiple pathologies and multimorbidity patterns are topics of increased interest as the world’s population ages. To explore the impact of multimorbidity on affected patients and the consequences for health services, we designed a study to describe multimorbidity by sex and life-stage in a large population sample and to assess the association with acute morbidity, area of residency and use of health services.

**Methods:**

A cross-sectional study was conducted in Catalonia (Spain). Participants were 1,749,710 patients aged 19+ years (251 primary care teams). Primary outcome: Multimorbidity (≥2 chronic diseases). Secondary outcome: Number of new events of each acute disease. Other variables: number of acute diseases per patient, sex, age group (19–24, 25–44, 45–64, 65–79, and 80+ years), urban/rural residence, and number of visits during 2010.

**Results:**

Multimorbidity was present in 46.8% (95% CI, 46.7%-46.8%) of the sample, and increased as age increased, being higher in women and in rural areas. The most prevalent pair of chronic diseases was hypertension and lipid disorders in patients older than 45 years. Infections (mainly upper respiratory infection) were the most common acute diagnoses. In women, the highest significant RR of multimorbidity vs. non-multimorbidity was found for teeth/gum disease (aged 19–24) and acute upper respiratory infection. In men, this RR was only positive and significant for teeth/gum disease (aged 65–79). The adjusted analysis showed a strongly positive association with multimorbidity for the oldest women (80+ years) with acute diseases and women aged 65–79 with 3 or more acute diseases, compared to patients with no acute diseases (OR ranged from 1.16 to 1.99, p < 0.001). Living in a rural area was significantly associated with lower probability of having multimorbidity. The odds of multimorbidity increased sharply as the number of visits increased, reaching the highest probability in those aged 65–79 years.

**Conclusions:**

Multimorbidity is related to greater use of health care services and higher incidence of acute diseases, increasing the burden on primary care services. The differences related to sex and life-stage observed for multimorbidity and acute diseases suggest that further research on multimorbidity should be stratified according to these factors.

## Background

Concurrent diseases, multiple pathologies and multimorbidity patterns are topics of increased interest as the world’s population ages [1]. Multimorbidity is the coexistence of two or more chronic health problems in the same person at one point in time [2], and multimorbidity patterns are any combination of chronic diseases [3]. Both considerations have important consequences for the individual and for health services [4]. Multimorbidity is a challenge for industrialized countries and can jeopardize the viability of national health systems.

Traditionally, the construct of multimorbidity has been inherently associated with persistent or chronic disease. Methods to measure multimorbidity include disease scores, case-mix systems, indexes and disease counts, the latter being the common method [5]. Far too little attention has been paid to the use of health services and the role of urban or rural residency in patients with multimorbidity [6,7]. Furthermore, the literature lacks comparisons by sex of acute morbidity in patients with multimorbidity in a large population sample [8,9].

The classification of acute and chronic disease remains controversial. Acute disease is characterized by a single or repeated episode of relatively rapid onset and short duration with a recovery to previous stage of activity [10]. Nevertheless, some diseases fall into a grey area. Knowledge of specific acute diseases that may occur more frequently than expected and of the underlying vulnerabilities [11] could help to focus attention on the patients with multimorbidity rather than emphasizing the diseases.

To explore the impact of multimorbidity on affected patients and the consequences for health services, we designed a study to describe multimorbidity by sex and life-stage in a large population sample and to assess the association with acute morbidity, area of residency and use of health services.

## Methods

### Data source and study population

Cross-sectional study of adults resident in Catalonia, a Mediterranean region of southern European with 7,434,632 inhabitants (2010 census), 16% of the population of Spain. In Catalonia, 358 primary health care teams (PHCT) comprised of doctors, nurses, social workers and support staff are assigned by geographical area and responsible for the health care of the population in their areas. The Catalan Health Institute (CHI) manages 274 PHCT (76.5%), serving a population of 4,859,725 adults; the remaining PHCT are managed by other providers. Doctors and nurses systematically use electronic health records (EHR) to record diagnoses, prescriptions and other clinical, patient management and administrative activities. The CHI Information System for the Development of Research in Primary Care (SIDIAP) [12] compiles coded clinical information from the EHR system. A subset of SIDIAP records meeting the highest quality criteria for clinical data (SIDIAP-Q) includes 40% of the SIDIAP population (1,936,443 patients), attended by the 1,319 general practitioners (GP) whose data recording scored highest in a validated comparison process. The sample is representative of the general Catalan population in terms of geography, age and sex distributions, according to the official 2010 census [13].

A sample of 1,749,710 patients aged 19 years or older, assigned to 251 PHCT during the period of study (1 January- 31 December 2010), was selected from the SIDIAP-Q database.

### Coding of diseases

International Classification of Diseases (ICD-10) codes were mapped to the International Classification of Primary Care (ICPC-2e-v.4.2, available at: http://www.kith.no/templates/kith_WebPage____1111.aspx). R codes (symptoms, signs and abnormal clinical and laboratory findings, not elsewhere classified) and Z codes (factors influencing health status and contact with health services) were excluded, resulting in 686 included codes. Each diagnosis was then classified using O’Halloran criteria for chronic disease [14]. We included all 146 diagnoses considered as chronic diseases by O’Halloran criteria: (i) have a duration that has lasted, or is expected to last, at least 6 months; (ii) have a pattern of recurrence or deterioration; (iii) have a poor prognosis and (iv) produce consequences, or sequelae, that have an impact on the individual’s quality of life [14,15]. Any disease not meeting the O’Halloran criteria was considered an acute disease.

All results were described with ICPC-2 codes. Diseases were classified as acute if diagnosed during the study period and chronic if recorded as such in EHR as of 31 December 2010.

### Outcomes and variables

The main outcome was multimorbidity, defined as the coexistence of 2 or more chronic diseases. Secondary outcome was the number of new events of each acute disease. Other variables recorded for each patient were the following: number of all acute diseases (0, 1, 2, > = 3), sex (male, female), age (young adult, 19 to 24; adult, 25–44; older adult, 45–64; elderly, 65–79; and oldest adult, 80+), number of visits during the study period (0, 1–2, 3–5, 6–10, ≥11), and area of residence (rural if <10,000 inhabitants and/or population density <150 people/km^2^, otherwise urban) [14]. Number of all acute diseases (or 0 diseases) and visits (or 0 visits) were categorized as quartiles of the study population. Number of visits was used as a proxy of use of health services and included visits recorded in EHR by GP, nurses or social workers, either at the primary care centre or as home health care.

### Statistical analysis

Analysis was stratified by sex and age group. Descriptive statistics were used to summarize overall information. Categorical variables were expressed as frequencies (percentage) and continuous as mean (Standard deviation, SD) or median (interquartile range, IQR).

Cumulative incidence of acute morbidity events was calculated as the number of new acute events during the study period divided by the at-risk population in the sample (e.g., if a patient had bronchitis twice in the one-year study period, the total number of events accounted for was 2). We took into account the five acute diseases with the highest cumulative incidence within each stratum. Risk ratios (RR) of multimorbidity vs. non-multimorbidity were calculated for the number of events for each acute disease, using Poisson, negative binomial (if overdispersion was present) or zero inflated (when data had an excess of zero counts) equations, as appropriate. All models were adjusted for number of visits and area of residency.

To determine the most prevalent multimorbidity patterns, all possible combinations of any two chronic diseases and their frequencies were calculated. Observed (O) and expected (E) prevalence of those two chronic diseases with each acute disease was then computed. Expected co-occurrence of diseases was obtained as the product of these prevalences, assuming statistical independence of the diseases concerned. The overlapping of those combinations that presented the highest O/E ratio was reported.

Logistic regression was used to assess the association between multimorbidity and the variables listed above.

All statistical tests were two-sided at the 5% significance level. The analyses were performed using SPSS for Windows, version 18 (SPSS Inc., Chicago, IL, USA), Stata/SE version 11 for Windows (Stata Corp. LP, College Station, TX, USA) and R version 2.15.2 (R Foundation for Statistical Computing, Vienna, Austria).

### Ethical considerations

The study protocol was approved by the Committee on the Ethics of Clinical Research, Institut Universitari d’Investigació en Atenció Primària (IDIAP) Jordi Gol (Protocol No: P12/28). All data were anonymized and the confidentiality of EHR was respected at all times in accordance with international law.

## Results

We included 1,749,710 patients; mean age was 47.4 years (SD: 17.8), 50.7% were female and 16% lived in rural areas. Multimorbidity (≥ 2 diseases) was present in 46.8% (95%CI, 46.7%-46.8%) of the sample, being higher in female (52.3%) than in male (41.1%) and in rural areas (47.6%) than in urban areas (46.6%).

The prevalence of the most common chronic diseases differed by sex below 45: anxiety disorder/anxiety (women aged 19–44); acne (men aged 19–24) and lipid disorder (men aged 25–44). After 45 both sex groups first chronic disease was lipid disorder in 45–64 and uncomplicated hypertension in 65-80+ (Table 1). Upper respiratory infection acute is the most incident acute disease in all age groups (except in 80+).

**Table 1:** Five highest cumulative incidence of acute and prevalence of chronic diseases by sex and age groups

**Female**							**Male**					
**Age groups**	**ICPC**	**Chronic diseases**	**Prevalence (%, CI)**	**ICPC**	**Acute diseases**	**Cumulative incidence (%, CI)**	**ICPC**	**Chronic diseases**	**Prevalence (%, CI)**	**ICPC**	**Acute diseases**	**Cumulative incidence (%, CI)**
19-24	P74	Anxiety disorder/anxiety state	8.4 (8.2-8.7)	R74	Upper respiratory infection acute	9.1 (8.9-9.3)	S96	Acne	7.7 (7.5-7.9)	R74	Upper respiratory infection acute	6.9 (6.7-7.1)
	S96	Acne	7.8 (7.6-8.0)	R76	Tonsillitis acute	4.4 (4.3-4.6)	R96	Asthma	6.0 (5.8-6.2)	R76	Tonsillitis acute	3.4 (3.3-3.6)
	R96	Asthma	5.4 (5.2-5.6)	U71	Cystitis/urinary infection other	4.1 (4.0-4.3)	P74	Anxiety disorder/anxiety state	3.9 (3.7-4.0)	D73	Gastroenteritis presumed infection	2.9 (2.8-3.0)
	T82	Obesity	5.0 (4.8-5.1)	D82	Teeth/gum disease	3.7 (3.5-3.8)	T82	Obesity	3.4 (3.3-3.6)	D82	Teeth/gum disease	2.5 (2.4-2.7)
	L85	Acquired deformity of spine	4.6 (4.4-4.8)	D73	Gastroenteritis presumed infection	3.6 (3.5-3.8)	L85	Acquired deformity of spine	3.1 (3.0-3.2)	S16	Bruise/contusion	2.3 (2.2-2.4)
25-44	P74	Anxiety disorder/anxiety state	12.2 (12.1-12.3)	R74	Upper respiratory infection acute	7.8 (7.7-7.9)	T93	Lipid disorder	7.3 (7.2-7.4)	R74	Upper respiratory infection acute	5.9 (5.8-5.9)
	P76	Depressive disorder	8.8 (8.7-8.9)	L03	Low back symptom/complaint	3.4 (3.3-3.5)	P74	Anxiety disorder/anxiety state	6.5 (6.4-6.6)	D73	Gastroenteritis presumed infection	2.4 (2.4-2.5)
	T82	Obesity	6.8 (6.7-6.9)	U71	Cystitis/urinary infection other	2.9 (2.8-2.9)	T82	Obesity	4.4 (4.4-4.5)	L03	Low back symptom/complaint	2.4 (2.3-2.4)
	T93	Lipid disorder	5.0 (4.9-5.0)	D73	Gastroenteritis presumed infection	2.8 (2.8-2.9)	P76	Depressive disorder	3.7 (3.7-3.8)	D82	Teeth/gum disease	2.1 (2.1-2.2)
	N89	Migraine	4.9 (4.8-4.9)	R76	Tonsillitis acute	2.6 (2.6-2.7)	L86	Back syndrome with radiating pain	3.5 (3.4-3.5)	R76	Tonsillitis acute	1.8 (1.8-1.9)
45-64	T93	Lipid disorder	28.4 (28.2-28.5)	R74	Upper respiratory infection acute	7.0 (6.9-7.1)	T93	Lipid disorder	29.9 (29.7-30.1)	R74	Upper respiratory infection acute	4.8 (4.7-4.9)
	K86	Hypertension uncomplicated	21.2 (21.1-21.4)	L03	Low back symptom/complaint	3.2 (3.1-3.3)	K86	Hypertension uncomplicated	24.6 (24.4-24.7)	L03	Low back symptom/complaint	2.6 (2.5-2.7)
	P76	Depressive disorder	18.9 (18.8-19.1)	U71	Cystitis/urinary infection other	2.9 (2.8-3.0)	T82	Obesity	10.9 (10.8-11.0)	R78	Acute bronchitis/bronchiolitis	2.0 (2.0-2.1)
	T82	Obesity	15.7 (15.6-15.9)	R78	Acute bronchitis/bronchiolitis	2.8 (2.7-2.9)	T90	Diabetes non-insulin dependent	10.3 (10.2-10.5)	D82	Teeth/gum disease	2.0 (2.0-2.1)
	P74	Anxiety disorder/anxiety state	13.5 (13.4-13.6)	L20	Joint symptom/complaint NOS	2.6 (2.6-2.7)	L86	Back syndrome with radiating pain	7.6 (7.5-7.7)	H81	Excessive ear wax	1.9 (1.9-2.0)
65-79	K86	Hypertension uncomplicated	60.3 (60.0-60.6)	R74	Upper respiratory infection acute	7.2 (7.1-7.3)	K86	Hypertension uncomplicated	56.2 (55.9-56.5)	R74	Upper respiratory infection acute	6.4 (6.2-6.5)
	T93	Lipid disorder	52.4 (52.1-52.7)	U71	Cystitis/urinary infection other	4.3 (4.2-4.4)	T93	Lipid disorder	44.6 (44.3-44.9)	H81	Excessive ear wax	3.9 (3.8-4.0)
	T82	Obesity	24.9 (24.7-25.1)	R78	Acute bronchitis/bronchiolitis	3.8 (3.7-3.9)	Y85	Benign prostatic hypertrophy	28.4 (28.1-28.7)	R78	Acute bronchitis/bronchiolitis	3.5 (3.4-3.6)
	L95	Osteoporosis	22.8 (22.5-23.0)	H81	Excessive ear wax	3.0 (2.9-3.1)	T90	Diabetes non-insulin dependent	25.6 (25.4-25.9)	D82	Teeth/gum disease	2.4 (2.3-2.5)
	P76	Depressive disorder	22.3 (22.1-22.5)	L03	Low back symptom/complaint	2.8 (2.7-2.9)	T82	Obesity	15.4 (15.2-15.6)	L03	Low back symptom/complaint	2.3 (2.2-2.4)
80+	K86	Hypertension uncomplicated	73.1 (72.7-73.4)	R74	Upper respiratory infection acute	5.1 (4.9-5.2)	K86	Hypertension uncomplicated	63.4 (62.9-63.9)	H81	Excessive ear wax	5.8 (5.6-6.1)
	T93	Lipid disorder	44.5 (44.1-44.9)	U71	Cystitis/urinary infection other	5.0 (4.9-5.2)	Y85	Benign prostatic hypertrophy	37.3 (36.8-37.8)	R74	Upper respiratory infection acute	5.4 (5.1-5.6)
	L91	Osteoarthrosis other	25.7 (25.4-26.1)	R78	Acute bronchitis/bronchiolitis	4.5 (4.4-4.7)	T93	Lipid disorder	35.0 (34.5-35.5)	R78	Acute bronchitis/bronchiolitis	4.9 (4.7-5.2)
	F92	Cataract	23.5 (23.2-23.8)	H81	Excessive ear wax	4.2 (4.0-4.4)	T90	Diabetes non-insulin dependent	25.4 (24.9-25.9)	S18	Laceration/cut	3.8 (3.6-4.0)
	T90	Diabetes non-insulin dependent	22.8 (22.5-23.1)	S18	Laceration/cut	3.4 (3.3-3.6)	F92	Cataract	21.9 (21.4-22.3)	U71	Cystitis/urinary infection other	2.7 (2.6-2.9)

*Abbreviations:* ICPC 2 International Classification of Primary Care, CI Confidence interval.

Multimorbidity prevalence increased as age increased, being higher in female (ranged from 19.0% to 92.1%) than male (ranged from 12.9% to 92.0%). In patients with multimorbidity, the number of acute diseases was higher in female than male and decreased as age increased, except in male older than 65. In addition, the number of visits increased as age increased, and was higher for female than male in all age groups except 80+ (Table 2).

**Table 2:** Multimorbidity prevalence and acute diseases, area of residency and visits according to multimorbidity status stratified by sex and age groups

	**Female**									
	**19-24**		**25-44**		**45-64**		**65-79**		**≥80**	
	**MM**	**Non-MM**	**MM**	**Non-MM**	**MM**	**Non-MM**	**MM**	**Non-MM**	**MM**	**Non-MM**
	**n = 12,804 (19.0%)**	**n = 54,700 (81.0%)**	**n = 105,463 (29.4%)**	**n = 253,771 (70.6%)**	**n = 167,778 (63.3%)**	**n = 97,089 (36.7%)**	**n = 119,528 (90.2%)**	**n = 13,021 (9.8%)**	**n = 58,533 (92.1%)**	**n = 5,021 (7.9%)**
Number of acute diseases										
*Median (IQR)*	*1 (0–2)*	*0 (0–1)*	*1 (0–2)*	*0 (0–1)*	*1 (0-2)*	*0 (0–1)*	*1 (0–1)*	*0 (0–0)*	*0 (0–1)*	*0 (0–0)*
0	36.4	55.3	40.2	60.3	44.8	68.2	47.7	78.0	51.5	82.3
1	26.8	23.7	27.1	22.3	27.6	19.4	27.4	14.9	26.2	12.3
2	16.9	11.6	16.1	10.2	14.7	7.8	13.7	4.9	12.7	3.7
≥3	19.9	9.4	16.6	7.2	12.9	4.6	11.2	2.3	9.7	1.8
Living in a rural area	14.4	14.9	15.3	15.1	15.4	16.7	15.4	17.6	18.4	18.7
Number of visits										
*Median (IQR)*	*6 (3–10)*	*2 (0–5)*	*6 (3–11)*	*2 (0–5)*	*8 (4–13)*	*2 (0–5)*	*11 (7–18)*	*2 (0–5)*	*14 (8–24)*	*1 (0–7)*
0	8.8	28.4	9.8	34.2	5.8	38.3	2.6	38.9	3.0	40.4
1-2	15.4	24.4	14.7	22.9	10.4	21.6	4.4	18.6	4.2	16.2
3-5	25.0	23.6	23.1	21.5	20.5	20.1	11.9	18.0	9.1	14.0
6-10	27.1	15.8	26.7	14.5	28.7	13.5	26.5	15.5	20.4	13.4
≥11	23.6	7.8	25.7	6.9	34.6	6.4	54.7	9.0	63.4	16.0
	**Male**									
	**19-24**		**25-44**		**45-64**		**65-79**		**≥80**	
	**MM**	**Non-MM**	**MM**	**Non-MM**	**MM**	**Non-MM**	**MM**	**Non-MM**	**MM**	**Non-MM**
	**n = 8,916 (12.9%)**	**n = 60,373 (87.1%)**	**n = 75,556 (19.5%)**	**n = 311,124 (80.5%)**	**n = 139,776 (54.0%)**	**n = 119,008 (46.0%)**	**n = 97,044 (86.9%)**	**n = 14,584 (13.1%)**	**n = 32,773 (92.0%)**	**n = 2,948 (8.0%)**
Number of acute diseases										
*Median (IQR)*	*1 (0–1)*	*0 (0–1)*	*0 (0–1)*	*0 (0–1)*	*0 (0–1)*	*0 (0–1)*	*0 (0–1)*	*0 (0–0)*	*0 (0–1)*	*0 (0–0)*
0	49.0	64.6	50.8	68.1	55.3	74.0	53.2	76.4	52.0	78.9
1	26.1	21.7	26.4	20.0	26.0	17.3	26.9	16.0	27.1	13.7
2	13.7	8.4	12.8	7.6	11.3	5.9	12.0	5.3	11.9	4.6
≥3	11.1	5.2	10.0	4.4	7.4	2.8	8.0	2.3	8.9	2.8
Living in a rural area	15.0	15.1	15.6	15.3	17.3	17.5	16.7	18.8	21.3	21.5
Number of visits										
*Median (IQR)*	*4 (1–8)*	*1 (0–4)*	*5 (2–9)*	*1 (0–3)*	*6 (3–12)*	*1 (0–3)*	*11 (6–17)*	*2 (0–6)*	*14 (8–24)*	*2 (0–8)*
0	15.0	38.7	14.2	43.8	8.9	46.1	2.8	36.0	2.3	35.2
1-2	21.6	26.3	18.4	24.0	13.4	21.7	5.3	19.4	3.5	16.3
3-5	26.1	19.8	24.4	18.0	22.1	17.1	13.4	18.8	8.8	15.5
6-10	22.5	10.8	23.2	10.0	26.9	10.3	28.1	16.1	20.8	14.2
≥11	14.8	4.4	19.8	4.2	28.7	4.7	50.4	9.7	64.5	18.8

*Abbreviations:* MM multimorbidity, IQR interquartile range. Data are expressed as percentage, unless otherwise stated.

Patients with multimorbidity had a higher incidence of acute diseases and number of visits in all age strata than non-multimorbidity patients; in both cases, the incidence was higher for female than male (Table 2). Overall, the median (IQR) of number of visits was 8(4–14) in patients with multimorbidity vs. 1(0–4) in the non-multimorbidity group.

**Figure 1:**
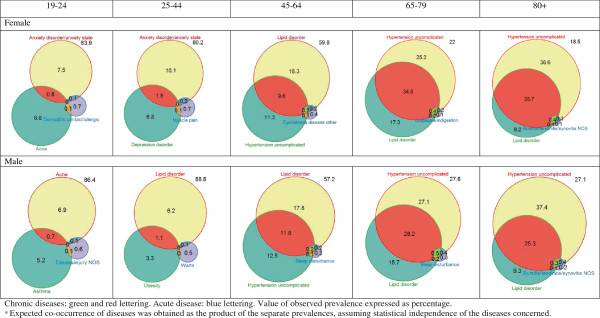
Most prevalent multimorbidity patterns of two chronic diseases and the corresponding acute disease with the highest observed/expected ratio^*^, by sex and age groups.

The two most prevalent combinations of two chronic diseases were hypertension and lipid disorders in patients older than 45 years. The only acute disease that appeared in both sexes was “bursitis/tendinitis/synovitis NOS” in the oldest age group (80+). In the other age groups, the acute health disease varied by sex (Figure 1).

The five acute diseases with the highest cumulative incidence were similar by sex in any age group. Infections were the most common diagnosis. Cystitis/urinary infection was present among the five most prevalent acute conditions only in women and in the oldest men. In women, the highest significant RR of multimorbidity vs. non-multimorbidity was found for teeth/gum disease (aged 19–24) and upper respiratory infection, acute (80+). In men, this RR was only positive and significant for teeth/gum disease (aged 65–79) (Table 3).

**Table 3:** Cumulative incidence and risk ratio (RR) of multimorbidity for the five acute diseases with the highest cumulative incidence by sex and age groups*^#^

**Female**							**Male**					
**Age groups**	**ICPC 2 code**	**Acute diseases**	**MM CI**	**Non-MM CI**	**Risk ratio**	**95% confidence interval**	**ICPC 2 code**	**Acute diseases**	MM CI*	**Non-MM CI**	**Risk ratio**	**95% confidence interval**
			**(%)**	**(%)**					**(%)**	**(%)**		
19-24	R74	Upper respiratory infection, acute	16.2	8.8	**1.10**	**(1.04-1.16)**	R74	Upper respiratory infection, acute	11.9	7.0	0.96	(0.89-1.03)
	R76	Tonsillitis, acute	6.7	4.3	0.94	(0.86-1.02)	R76	Tonsillitis, acute	5.5	3.4	0.92	(0.83-1.02)
	U71	Cystitis/urinary infection, other	6.3	4.1	**0.85**	**(0.78-0.93)**	D73	Gastroenteritis, presumed infection	4.8	2.8	0.93	(0.83-1.04)
	D82	Teeth/gum disease	6.2	3.6	**1.14**	**(1.04-1.25)**	D82	Teeth/gum disease	4.3	2.7	**0.63**	**(0.44-0.89)**
	D73	Gastroenteritis, presumed infection	6.3	3.4	1.06	(0.97-1.16)	S16	Bruise/contusion	3.2	2.4	**0.75**	**(0.66-0.86)**
25-44	R74	Upper respiratory infection, acute	12.8	6.9	1.01	(0.98-1.03)	R74	Upper respiratory infection, acute	9.9	5.7	**0.85**	**(0.82-0.87)**
	L03	Low back symptom/complaint	4.4	3.1	**0.73**	**(0.71-0.76)**	D73	Gastroenteritis, presumed infection	3.8	2.3	**0.79**	**(0.76-0.83)**
	U71	Cystitis/urinary infection other	4.4	2.6	**0.86**	**(0.83-0.90)**	L03	Low back symptom/complaint	3.5	2.2	**0.75**	**(0.71-0.78)**
	D73	Gastroenteritis presumed infection	4.3	2.5	**0.90**	**(0.87-0.94)**	D82	Teeth/gum disease	3.5	2.2	**0.82**	**(0.72-0.93)**
	R76	Tonsillitis, acute	3.6	2.5	1.08	(0.91-1.29)	R76	Tonsillitis, acute	2.4	1.8	**0.69**	**(0.65-0.73)**
45-64	R74	Upper respiratory infection, acute	9.7	4.5	**0.92**	**(0.88-0.95)**	R74	Upper respiratory infection, acute	6.7	3.4	**0.79**	**(0.76-0.82)**
	L03	Low back symptom/complaint	3.9	2.1	**0.78**	**(0.74-0.82)**	L03	Low back symptom/complaint	3.2	2.1	**0.58**	**(0.55-0.62)**
	U71	Cystitis/urinary infection, other	4.0	1.7	**0.86**	**(0.81-0.92)**	R78	Acute bronchitis/bronchiolitis	3.0	1.3	**0.86**	**(0.80-0.92)**
	R78	Acute bronchitis/bronchiolitis	3.9	1.6	0.95	(0.89-1.01)	D82	Teeth/gum disease	2.9	1.6	**0.83**	**(0.70-0.98)**
	L20	Joint symptom/complaint NOS	3.3	1.5	0.97	(0.82-1.15)	H81	Excessive ear wax	2.5	1.4	**0.74**	**(0.69-0.78)**
65-79	R74	Upper respiratory infection, acute	8.4	3.2	0.92	(0.70-1.20)	R74	Upper respiratory infection, acute	7.6	3.3	0.93	(0.84-1.03)
	U71	Cystitis/urinary infection other	5.1	1.4	1.09	(0.91-1.30)	H81	Excessive ear wax	4.4	2.3	**0.74**	**(0.65-0.83)**
	R78	Acute bronchitis/bronchiolitis	4.5	1.3	0.71	(0.36-1.39)	R78	Acute bronchitis/bronchiolitis	4.2	1.5	0.99	(0.85-1.14)
	H81	Excessive ear wax	3.3	1.2	0.88	(0.74-1.04)	D82	Teeth/gum disease	2.9	1.2	**1.31**	**(1.10-1.57)**
	L03	Low back symptom/complaint	3.0	1.1	0.76	(0.54-1.07)	L03	Low back symptom/complaint	2.5	1.1	0.87	(0.73-1.03)
80+	R74	Upper respiratory infection, acute	5.8	1.5	**1.47**	**(1.16-1.86)**	H81	Excessive ear wax	6.5	2.7	0.98	(0.77-1.24)
	U71	Cystitis/urinary infection, other	5.9	1.5	**1.40**	**(1.10-1.78)**	R74	Upper respiratory infection, acute	6.2	2.0	1.29	(0.98-1.70)
	R78	Acute bronchitis/bronchiolitis	5.3	2.0	0.99	(0.80-1.23)	R78	Acute bronchitis/bronchiolitis	5.7	2.0	1.06	(0.80-1.40)
	H81	Excessive ear wax	4.7	1.3	**1.34**	**(1.04-1.73)**	S18	Laceration/cut	4.3	2	**0.75**	**(0.57-0.99)**
	S18	Laceration/cut	3.9	1.6	**0.78**	**(0.61-0.99)**	U71	Cystitis/urinary infection, other	3.0	1.5	0.73	(0.53-1.01)

*Abbreviations:* MM multimorbidity, RR risk ratio, ICPC 2 International Classification of Primary Care, CI Cumulative incidence, NOS not otherwise specified.

*Cumulative incicidence was calculated as the sum of the number of new acute events during the study period divided by the 2010 population (e.g. if a patient passed two bronchitis in one year. the total number of events accounted for was two).

^#^The outcome is the number of acute diseases in each person for each acute disease considered. RR of multimorbidity versus non-multimorbidity was calculated by Poisson, negative binomial (if overdispersion is present) or zero inflated (when data showed excess of zero counts) equation as appropriate and adjusted for number of visits and area of residency. In bold P < 0.05.

The adjusted analysis of factors associated with multimorbidity showed that the oldest patients (80+ years) with acute diseases and women aged 65–79 with 3 or more acute diseases were more likely to have multimorbidity than patients with no acute diseases. This positive association was only significant in women. Living in a rural area was significantly associated with lower probability of having multimorbidity. Patients who visited a GP more often were more likely than those without visits to have multimorbidity, reaching the highest probability in those aged 65–79 years (Table 4).

**Table 4:** Factors associated with multimorbidity by sex and age groups

**Female**															
	**19-24**			**25-44**			**45-64**			**65-79**			**80+**		
	**OR**	**95% CI**	**P-value**	**OR**	**95% CI**	**P-value**	**OR**	**95% CI**	**P-value**	**OR**	**95% CI**	**P-value**	**OR**	**95% CI**	**P-value**
*Number of acute diseases(ref.0)*			0.030			<0.001			<0.001			<0.001			<0.001
1	0.94	0.89-0.99		0.88	0.86-0.90		0.82	0.80-0.84		0.98	0.92-1.03		1.16	1.05-1.28	
2	0.95	0.89-1.01		0.86	0.83-0.88		0.76	0.73-0.78		1.05	0.96-1.15		1.54	1.31-1.80	
≥3	1.02	0.95-1.09		0.89	0.87-0.92		0.78	0.75-0.81		1.29	1.14-1.46		1.99	1.59-2.47	
*Rural area (ref. urban)*	0.94	0.89-0.99	0.025	0.94	0.92-0.96	<0.001	0.80	0.78-0.82	<0.001	0.69	0.65-0.73	<0.001	0.76	0.70-0.83	<0.001
*Number of visits (ref. 0)*			<0.001			<0.001			<0.001			<0.001			<0.001
1-2	2.09	1.93-2.26		2.34	2.28-2.14		3.40	3.30-3.51		3.62	3.38-3.87		3.41	3.08-3.78	
3-5	3.52	3.25-3.80		4.04	3.93-4.15		7.54	7.31-7.77		10.18	9.54-10.86		8.36	7.55-9.27	
6-10	5.69	5.25-6.17		7.11	6.91-7.32		16.28	15.76-16.81		25.98	24.28-27.80		18.86	16.99-20.94	
≥11	9.93	9.09-10.85		14.52	14.05-14.99		43.03	41.39-44.73		90.81	83.74-98.48		45.19	40.69-50.19	
**Male**															
	**19-24**			**25-44**			**45-64**			**65-79**			**80+**		
	**OR**	**95% CI**	**P-value**	**OR**	**95% CI**	**P-value**	**OR**	**95% CI**	**P-value**	**OR**	**95% CI**	**P-value**	**OR**	**95% CI**	**P-value**
*Number of acute diseases(ref.0)*			<0.001			<0.001			<0.001			<0.001			0.590
1	0.78	0.73-0.83		0.71	0.70-0.73		0.67	0.65-0.69		0.84	0.80-0.89		1.07	0.95-1.21	
2	0.79	0.73-0.86		0.64	0.62-0.66		0.58	0.56-0.60		0.78	0.72-0.85		1.08	0.89-1.31	
≥3	0.76	0.69-0.83		0.60	0.58-0.62		0.55	0.52-0.57		0.86	0.77-0.97		1.12	0.88-1.42	
*Rural area (ref. urban)*	0.94	0.88-1.00	0.045	0.95	0.93-0.97	<0.001	0.87	0.85-0.89	<0.001	0.71	0.68-0.75	<0.001	0.79	0.71-0.88	<0.001
*Number of visits (ref. 0)*			<0.001			<0.001			<0.001			<0.001			<0.001
1-2	2.32	2.15-2.50		2.67	2.60-2.75		3.57	3.47-3.67		3.58	3.35-3.82		3.33	2.89-3.85	
3-5	3.93	3.64-4.25		5.18	5.04-5.33		8.20	7.97-8.43		9.66	9.06-10.30		8.62	7.50-9.92	
6-10	6.51	5.97-7.09		9.62	9.33-9.92		17.78	17.24-18.34		24.51	22.93-26.19		22.07	19.13-25.47	
≥11	10.86	9.82-12.01		20.58	19.86-21.32		45.13	43.43-46.90		76.41	70.68-82.60		51.32	44.49-59.19	

Logistic regression.

*Abbreviations:* OR odds ratio, CI confidence interval, ref. reference.

## Discussion

### Statement of principal findings

Almost half of the study population had multimorbidity, with infections (mainly acute upper respiratory infection) the most common acute disease in both sexes and all age groups. The most frequent multimorbidity pattern of chronic diseases was the combination of hypertension and dyslipidemia in adults over 45 years of age.

We observed a decrease in the number of acute diseases recorded as age increased. Nonetheless, in adjusted models female older than 65 who had acute diseases were more likely to have multimorbidity.

Finally, the use of health services was positively associated with a diagnosis of multimorbidity. Living in a rural area decreased the probability of multimorbidity.

### Strengths and weaknesses of the study

A major strength of this study is the analysis of a large, high-quality database of primary-care records, representative of a large population. In the context of a national health system with universal coverage, EHR data have been shown to yield more reliable and representative conclusions than those derived from survey-based studies [16]. Another important strength was the inclusion of all chronic and acute diagnoses registered in EHR, which contributed to a more accurate analysis of the association between acute and chronic diseases and of the disease combinations present in multimorbidity in this population. To synthesize the results, we present here only the most frequent combinations. Finally, few studies have incorporated acute diseases in the study of multimorbidity patterns [8] and none analyzed the relationship between multimorbidity and acute morbidity.

Some possible biases could have influenced our results. First, diseases could be underreported, especially for male of normal workforce age who tend to see their doctors less often than other strata of patients. This effect would diminish in the two oldest age groups because of the retirement age (65 years) in Spain. In patients with multimorbidity, the true incidence of acute disease could be underreported because the GP would place higher priority on the chronic problems in these patients. On the other hand, there could be an over-representation of chronic diagnoses (e.g., hypertension, diabetes, hyperlipidemia, etc.) that are included in the goals/incentives contracts of Catalan PHCT.

The diseases that form part of the CatSalut treatment objectives may be more carefully recorded than other conditions. However, these same diseases are the most prevalent (high blood pressure, diabetes, hypercholesterolemia, smoking, dyslipidemia, ischemic heart disease, atrial fibrillation) and therefore have the greatest impact on population health. The quality-recorders database (SIDIAP-Q) used in this study minimizes the under-reporting of diseases not included in the CatSalut objectives.

Furthermore, the stratified analysis allows more accurate estimation within each age-sex strata and universal access to free health care and medications makes it more likely that patients seeking care will acquire a diagnosis, either acute or chronic [17,18]. Second, there is no universally accepted criterion for consensus classification of acute and chronic disease. This lack of accurate case definitions impedes the establishment of the true incidence/prevalence of a disease [19]. Finally, a residual confounding cannot be completely excluded, and could occur because of epidemiological factors not considered in this study, such as patients’ socioeconomic status [20].

### Strengths and weaknesses in relation to other studies

The estimated multimorbidity prevalence in our sample is higher than in other European studies [21-24], perhaps because of the analysis of a greater number of diseases in our study than in most other published studies [25]. Nonetheless, the patterns of multimorbidity observed were similar to those observed in other studies [26].

As in other studies, multimorbidity was more prevalent among female [21,22,24]. This could be due to the longer female life expectancy and worse health status, compared to male, differences that are due to both biological and social factors [26]. In addition, sex is a social determinant that influences health status, health behaviours and the use of health services [27-30]. Recent studies, however, suggest a dismantling of this paradigm based on sex-stratified analysis of consultations for common symptoms [31].

Acute problems are time-consuming for health professional [32], and therefore should be considered part of the primary care workload. Although current health policy, health care services, and research are all heavily focused on chronic diseases, we must not forget that 41% of primary care visits are motivated by an acute disease [33]. The incidence of acute diseases observed in our study concurs with other reports of acute upper respiratory infection and other health problems of infectious aetiology (acute tonsillitis, cystitis) as the primary reasons for seeking primary care, along with non-infectious diseases such as dorsalgia [33,34].

Our study observed a higher prevalence of multimorbidity in rural settings. Other studies conducted in rural areas have reported only a greater prevalence of multimorbidity in elderly people [6,7]. Nonetheless, our adjusted analysis showed that living in a rural area is negatively associated with multimorbidity. This phenomenon could be due to the environmental and sociocultural context and access to both public and private services [28].

### Implications for clinicians and policymakers

Our study considered multimorbidity in patients who received primary medical care, considering all visits and diagnoses (acute and chronic diseases). This approach allowed the identification of vulnerable subgroups in our population. A major advantage of our methodology is the use of data obtained directly from standard clinical practice. Knowing the distribution of acute and chronic diseases by life-stage and sex will help the clinician faced with a particular patient to anticipate disease patterns based on the patient’s sex and stage of life, recognizing that these vary with age. This will encourage the implementation of personalized disease prevention and health promotion activities.

At the level of health policy and health care administration, the organization of services should be reviewed to ensure that continuity and coordination of patient care are guaranteed; current evidence suggests the potential for improvement in this regard [35].

### Unanswered questions and future research

The classification of chronic and acute disease remains unresolved, and there is no consensus on the type and number of chronic diseases that define multimorbidity. A personalized measure to determine the severity of diagnosed multimorbidity is also needed.

If longitudinal studies confirm a higher incidence of morbidity in patients with multimorbidity, evidence-based interventions will be needed to prevent the onset of acute disease. Further studies are needed to study possible genetic and pathophysiological explanations that corroborate the observed multimorbidity patterns.

In-depth analysis of other contextual factors related to multimorbidity is also required, along with studies of the relationship between area of residence and multimorbidity and of the differences in health status that may exist between different territories. Finally, there is a need for the implementation and evaluation of health literacy and self-management interventions to improve patient competence in resolving routine acute diseases, which in turn will decrease the care burden in primary care systems.

## Conclusions

Multimorbidity is related to more use of health services and higher incidence of acute diseases, which increases the burden on primary care services. Residence in urban vs rural settings is a factor for future in-depth study.

The association of acute morbidity, area of residency or use of health services with multimorbidity differs according to life-stage and sex. Therefore, the study of multimorbidity should be stratified by life-stage and sex. Understanding these trends across life-stages will allow health systems to adjust their clinical and management models to adapt and prioritize interventions.

## Abbreviations

PHCT: Primary health care teams; CHI: Catalan Health Institute; EHR: Electronic health records; SIDIAP: Information System for the Development of Research in Primary Care; GP: General practitioners; ICD: International Classification of Diseases; ICPC: International Classification of Primary Care; IDIAP: Institut Universitari d’Investigació en Atenció Primària; SD: Standard deviation; IQR: Interquartile range; RR: Risk ratios.

## Competing interests

The authors declare that they have no competing interests.

## Authors’ contributions

All authors contributed to the design of the study, revised the article, and approved the final version. CV, QFB, JMV, MMP, ARL drafted the study protocol and obtained the funding. TRB, CV, QFB, JMV, ARL contributed to the analysis and interpretation of data. CV, QFB, JMV, TRB, ARL, MPV, MMP, and EPR wrote the first draft, and all authors contributed ideas, interpreted the findings and reviewed rough drafts of the manuscript. All authors read and approved the final manuscript.

## Pre-publication history

The pre-publication history for this paper can be accessed here:

http://www.biomedcentral.com/1471-2296/15/55/prepub

## References

[B1] Bodenheimer T Wagner EH Grumbach K Improving primary care for patients with chronic illness JAMA 2002 288 1775 1779 12365965 10.1001/jama.288.14.1775

[B2] Van den Akker M Buntinx F Knottnerus JA Comorbidity or multimorbidity: what’s in a name? A review of literature Eur J Gen Pract 1996 2 65 70

[B3] Freund T Kunz CU Ose D Szecsenyi J Peters-Klimm F Patterns of multimorbidity in primary care patients at high risk of future hospitalization Popul Health Manag 2012 15 119 124 22313440 10.1089/pop.2011.0026

[B4] Gijsen R Hoeymans N Schellevis FG Ruwaard D Satariano WA van den Bos GA Causes and consequences of comorbidity: a review J Clin Epidemiol 2001 54 661 674 11438406 10.1016/s0895-4356(00)00363-2

[B5] van den Akker M Buntinx F Roos S Knottnerus JA Problems in determining occurrence rates of multimorbidity J Clin Epidemiol 2001 54 675 679 11438407 10.1016/s0895-4356(00)00358-9

[B6] Khanam MA Streatfield PK Kabir ZN Qiu C Cornelius C Wahlin A Prevalence and patterns of multimorbidity among elderly people in rural Bangladesh: a cross-sectional study J Health Popul Nutr 2011 29 406 414 21957680 10.3329/jhpn.v29i4.8458 PMC3190372

[B7] John R Kerby DS Hennessy CH Patterns and impact of comorbidity and multimorbidity among community-resident American Indian elders Gerontologist 2003 43 649 660 14570961 10.1093/geront/43.5.649

[B8] Saltman DC Sayer GP Whicker SD Co-morbidity in general practice Postgrad Med J 2005 81 474 480 15998827 10.1136/pgmj.2004.028530 PMC1743309

[B9] Broemeling A Watson D Black C Chronic conditions. Co-morbidity among residents of British Columbia 2005 Vancouver, BC: University of British Columbia

[B10] World Health Organization A Glossary of Terms for Community Health Care and Services for Older Persons 2004 Kobe: WHO

[B11] Wolff JL Starfield B Anderson G Prevalence, expenditures, and complications of multiple chronic conditions in the elderly Arch Intern Med 2002 162 2269 2276 12418941 10.1001/archinte.162.20.2269

[B12] Information system for the development of research in primary care (SIDIAP data base) [http://www.sidiap.org/]

[B13] García-Gil Mdel M Hermosilla E Prieto-Alhambra D Fina F Rosell M Ramos R Rodriguez J Williams T Van Staa T Bolibar B Construction and validation of a scoring system for the selection of high-quality data in a Spanish population primary care database (SIDIAP) Inform Prim Care 2011 19 3 135 145 22688222 10.14236/jhi.v19i3.806

[B14] O’Halloran J Miller GC Britt H Defining chronic conditions for primary care with ICPC-2 Fam Pract 2004 21 381 386 15249526 10.1093/fampra/cmh407

[B15] Defining Chronic Conditions for Primary Care Using ICPC-2 Available in: http://www.fmrc.org.au/Download/DefiningChronicConditions.pdf 10.1093/fampra/cmh407 15249526

[B16] Violán C Foguet-Boreu Q Hermosilla-Pérez E Valderas JM Bolíbar B Fàbregas-Escurriola M Brugulat-Guiteras P Muñoz-Pérez MÁ Comparison of the information provided by electronic health records data and a population health survey to estimate prevalence of selected health conditions and multimorbidity BMC Public Health 2013 13 251 23517342 10.1186/1471-2458-13-251 PMC3659017

[B17] Jordan K Porcheret M Croft P Quality of morbidity coding in general practice computerized medical records: a systematic review Fam Pract 2004 21 396 412 15249528 10.1093/fampra/cmh409

[B18] Valderas JM Starfield B Sibbald B Salisbury C Roland M Defining comorbidity: implications for understanding health and health services Ann Fam Med 2009 7 357 363 19597174 10.1370/afm.983 PMC2713155

[B19] de Lusignan S Tomson C Harris K van Vlymen J Gallagher H Creatinine fluctuation has a greater effect than the formula to estimate glomerular filtration rate on the prevalence of chronic kidney disease Nephron Clin Pract 2011 117 c213 c224 20805694 10.1159/000320341

[B20] Barnett K Mercer SW Norbury M Watt G Wyke S Guthrie B Epidemiology of multimorbidity and implications for health care, research, and medical education: a cros-sectional study Lancet 2012 380 37 43 22579043 10.1016/S0140-6736(12)60240-2

[B21] Prados-Torres A Poblador-Plou B Calderón-Larrañaga A Gimeno-Feliu LA González-Rubio F Poncel-Falcó A Sicras-Mainar A Alcalá-Nalvaiz JT Multimorbidity patterns in primary care: interactions among chronic diseases using factor analysis PLoS One 2012 7 e32190 22393389 10.1371/journal.pone.0032190 PMC3290548

[B22] Glynn LG Valderas JM Healy P Burke E Newell J Gillespie P Murphy AW The prevalence of multimorbidity in primary care and its effect on health care utilization and cost Fam Pract 2011 28 516 523 21436204 10.1093/fampra/cmr013

[B23] Salisbury C Johnson L Purdy S Valderas JM Montgomery AA Epidemiology and impact of multimorbidity in primary care: a retrospective cohort study Br J Gen Pract 2011 61 e12 e21 21401985 10.3399/bjgp11X548929 PMC3020068

[B24] García-Olmos L Salvador CH Alberquilla Á Lora D Carmona M García-Sagredo P Pascual M Muñoz A Monteagudo JL García-López F Comorbidity patterns in patients with chronic diseases in general practice PLoS One 2012 7 e32141 22359665 10.1371/journal.pone.0032141 PMC3281110

[B25] Marengoni A Angleman S Melis R Mangialasche F Karp A Garmen A Meinow B Fratiglioni L Aging with multimorbidity: a systematic review of the literature Ageing Res Rev 2011 10 430 439 21402176 10.1016/j.arr.2011.03.003

[B26] Laux G Kuehlein T Rosemann T Szecsenyi J Co- and multimorbidity patterns in primary care based on episodes of care: results from the German CONTENT project BMC Health Serv Res 2008 8 14 18205916 10.1186/1472-6963-8-14 PMC2244601

[B27] Lee JH Sadana R Improving Equity in Health by Addressing Social Determinants. The Commission on Social Determinants of Health Knowledge Networks 2011 World Health Organization [http://whqlibdoc.who.int/publications/2011/9789241503037_eng.pdf]

[B28] Marmot M Friel S Bell R Houweling TA Taylor S Commission on social determinants of health: closing the gap in a generation: health equity through action on the social determinants of health Lancet 2008 372 1661 1669 18994664 10.1016/S0140-6736(08)61690-6

[B29] Bertakis KD Azari R Helms LJ Callahan EJ Robbins JA Gender differences in the utilization of health care services J Fam Pract 2000 49 147 152 10718692

[B30] Redondo-Sendino A Guallar-Castillón P Banegas JR Rodríguez-Artalejo F Gender differences in the utilization of health-care services among the older adult population of Spain BMC Public Health 2006 6 155 16780576 10.1186/1471-2458-6-155 PMC1525176

[B31] Hunt K Adamson J Hewitt C Nazareth J Do women consult more than men? A review of gender and consultation for back pain and headache J Health Serv Res Policy 2011 16 108 117 20819913 10.1258/jhsrp.2010.009131 PMC3104816

[B32] Stange KC Zyzanski SJ Jaén CR Callahan EJ Kelly RB Gillanders WR Shank JC Chao J Medalie JH Miller WL Crabtree BF Flocke SA Gilchrist VJ Langa DM Goodwin MA Illuminating the ‘black box’. A description of 4454 patient visits to 138 family physicians J Fam Pract 1998 46 377 389 9597995

[B33] Woodwell DA Cherry DK National ambulatory medical care survey: 2002 summary Adv Data 2004 346 1 44 15460863

[B34] Wändell P Carlsson AC Wettermark B Lord G Cars T Ljunggren G Most common diseases diagnosed in primary care in Stockholm, Sweden, in 2011 Fam Pract 2013 506 13 10.1093/fampra/cmt033 23825186

[B35] Smith SM Soubhi H Fortin M Hudon C O’Dowd T Managing patients with multimorbidity: systematic review of interventions in primary care and community settings BMJ 2012 345 e5205 22945950 10.1136/bmj.e5205 PMC3432635

